# Association between baseline frailty and driving status over time: a secondary analysis of The National Health and Aging Trends Study

**DOI:** 10.1186/s40621-017-0106-y

**Published:** 2017-03-27

**Authors:** Elizabeth G. Bond, Laura L. Durbin, Jodi A. Cisewski, Min Qian, Jack M. Guralnik, Judith D. Kasper, Thelma J. Mielenz

**Affiliations:** 1 Department of Epidemiology, Columbia University Mailman School of Public Health, 722 West 168th Street, New York, NY 10032 USA; 2 Department of Biostatistics, Columbia University Mailman School of Public Health, New York, NY USA; 3 Division of Gerontology, Department of Epidemiology & Public Health, University of Maryland School of Medicine, Howard Hall Suite 200, 660 West Redwood Street, Baltimore, MD 21201 USA; 4 Department of Health Policy and Management, Johns Hopkins University, Bloomberg School of Public Health, 624 North Broadway, Baltimore, MD 21205 USA

**Keywords:** Driving, Epidemiology, Frailty, Older adults, Measurement

## Abstract

**Background:**

Continued automobile driving is important for the wellbeing and independence of older adults. Frailty has been associated with a variety of negative health outcomes, but studies are lacking on the potential association between frailty and driving status. The present study uses data from The National Health and Aging Trends Study (NHATS) to assess if the presence of frailty is associated with being a current non-driver.

**Methods:**

NHATS is a nationally representative cohort study of Medicare beneficiaries (aged ≥65) that have been followed since 2011. We examined frailty status at baseline (Fried’s frailty phenotype) and driving status over 4 years (from 2011 to 2014) excluding never drivers at baseline. Multivariable Poisson regression was used to obtain incidence rate ratios, adjusting for covariates and clustering. To account for the repeated measures in the data collection, generalized estimating equations (GEE) were employed.

**Results:**

A significant association between baseline frailty and driving status was observed at all four time points. At T4, frail participants at baseline had an incidence rate for becoming a current non-driver 1.80 times (or an 80% increase) that of non-frail participants at baseline (adjusted 95% confidence interval (CI) 1.56–2.07).

**Conclusions:**

Frailty was associated with an increased rate of being a current non-driver. Based on this association, we posit that screening for and intervening on frailty may help certain older adults who are at risk for becoming a current non-driver to remain on the road longer.

## Background

According to the United States Department of Transportation, a little more than 17% of the nation’s drivers were over the age of 65 in 2013 (Federal Highway Administration 2015). Keeping older adults driving who are appropriately safe on the road is important for their health and wellbeing, as driving cessation has been associated with poorer physical, social, and mental health outcomes among this population (Edwards et al. 2009a; O’Connor et al. 2013; Ragland et al. 2005). A review of the factors associated with safe driving behavior suggested a conceptual framework in which cognition, vision, and physical function determine an individual’s capacity to drive, and that actual capacity in combination with an individual’s beliefs about his or her own capacity determines driving behavior (Anstey et al. 2005). The authors further suggested that physical frailty as a separate concept may compound the effects of cognitive, visual, and physical functioning on driving capacity (Anstey et al. 2005). Although studies have examined the association between frailty and crash injuries, prospective studies of frailty and its associated risk of driving behaviors (including driving status) are lacking.

Frailty has been defined as being at increased vulnerability to stressors as a result of aging and the accumulation of multiple underlying physiological declines (Clegg et al. 2013; Fielding 2015; Fried et al. 2001). The most commonly used measure of frailty is the physical frailty phenotype proposed by Fried and colleagues in 2001 (Bouillon et al. 2013; Cesari et al. 2014; Fried et al. 2001; Walston & Bandeen-Roche 2015). The physical frailty phenotype consists of five criteria: weakness as measured by grip strength, self-reported exhaustion, unintentional weight loss (10 or more pounds in the past year), slow gait speed, and low physical activity (Fried et al. 2001). Frailty is defined as meeting three or more of the five criteria (Fried et al. 2001). This physical frailty phenotype has been shown to be a predictor of falls, worsening mobility, morbidity, and mortality (Fried et al. 2001). Our analysis uses measures collected by the National Health and Aging Trends Study (NHATS), which assessed frailty using the physical frailty phenotype. Our study evaluates the hypothesis that the presence of frailty will be associated with being a current non-driver, adjusting for lifestyle and clinical conditions that are associated with not driving.

## Methods

### Data source and study sample

This study used data from the National Health and Aging Trends Study (NHATS), a nationally representative, age-stratified longitudinal study of Medicare beneficiaries aged 65 and older who reside in the community in their own or another’s home or residential care setting (Montaquila, et al. 2012). Data have been collected annually since 2011. The study oversampled black individuals and individuals from older age groups. The same participants who were assessed in 2011 (T1) were also assessed in 2012 (T2), 2013 (T3), and 2014 (T4), allowing for longitudinal analyses to be conducted. Data were collected via 90 min in-person interviews conducted annually in participants’ homes. Physical and performance-based assessments, such as gait speed and grip strength, were obtained in addition to self-report data (Kasper & Freedman 2016). If a subject was unable to self-report data, a proxy respondent provided the information. During proxy interviews, subjects who were eligible for performance-based assessments had the opportunity to participate.

At baseline, 8,245 persons responded, with a response rate of 71% (Kasper & Freedman 2016). Our study sample consists of 6,288 persons who were in community or non-nursing home residential care settings, had a complete baseline frailty assessment, and were current or previous drivers at baseline. Never drivers (those who reported never having driven in their lifetime) were excluded from the study.

### Measures

#### Frailty

The physical frailty phenotype by Fried and colleagues consists of measures of weakness, slowness, exhaustion, low physical activity, and what Fried and colleagues labeled “shrinking” (Fried et al. 2001). Our frailty measure was based on information collected at the baseline time point (T1). We operationalized frailty following the approach of Bandeen-Roche and colleagues, in which the baseline (T1) NHATS data were used to assess the prevalence of frailty in the United States (Bandeen-Roche et al. 2015). In accordance with Bandeen-Roche and colleagues, weakness was defined as having a maximum grip strength over two trials that was at or below the 20th percentile and slowness was defined as having a first completed gait speed at or below the 20th percentile (Bandeen-Roche et al. 2015). Those who did not complete a grip or gait test for a medical reason were given a score of zero, indicating that they had failed that particular test. Exhaustion was defined by a self-report that the participant had “low energy or was easily exhausted: enough to limit their activities” at his or her baseline interview (Bandeen-Roche et al. 2015). Low physical activity was defined as self-report of not having recently walked for exercise or engaged in vigorous physical activity. Finally, shrinking was defined as having unintentionally lost 10 pounds or more in the past year or having a body mass index (BMI) of 18.5 kg/m^2^ or less. Those who were unable to be assessed for BMI because of a missing height or weight measurement were eliminated from the sample, as were those who were missing data for either both grip tests, both gait tests, either of the exhaustion questions, either of the low physical activity questions, or either of the weight loss questions.

All frailty categories were marked on a “one” (pass) or “zero” (fail) scale. An individual was considered to be frail (dichotomous outcome) if he or she failed in three or more of the five categories. Only data from those who had a complete baseline frailty assessment were used for the analysis.

#### Driving status

Driving status at T1 was assessed using the response to the question regarding the last time the participant had driven. Those community- or non-nursing home residential care-dwelling participants with frailty scores who responded that they had never driven in their lifetime (never drivers) (*n =* 541) or who reported they never left their residence or were blind (*n =* 81) were not included in the analysis (Skehan et al. 2014). At T1, our sample consisted of both current and former drivers. For the follow-up time points (T2-T4), driving status was assessed based on the participants’ responses to a question of how often they had driven in the last year: every day, most days, rarely, or never. Those who had never driven in the previous year were considered current non-drivers. Those who responded that they had driven rarely or more in the previous year were considered current drivers. It was possible for non-drivers at one time point to become resumed drivers at the next time point (e.g. after healing from a hip replacement), although this was rare. At T2 1.9% of the included sample were resumed drivers from T1, at T3 0.9% were resumed drivers from T2, and at T4 0.9% were resumed drivers from T3.

#### Covariates

Variables that were previously associated with driving cessation among older adults were included as covariates in the analyses, including gender, age, race/ethnicity, living arrangement, dementia, and a comorbidity scale (Anstey et al. 2006; Choi et al. 2013; Gwyther & Holland 2012; Herrmann et al. 2006). Gender was defined as male or female. Age was categorized as 65–69, 70–74, 75–79, 80–84, 85–89, and ≥90 years old. Race/ethnicity was defined as white non-Hispanic, black non-Hispanic, other non-Hispanic, and Hispanic. Living arrangement was defined as ether living alone in a household or not living alone in a household. Dementia was categorized into probable dementia and possible or no dementia. A description of this dementia categorization can be found elsewhere (Kasper et al. 2013). Individuals who had an unreported race/ethnicity or living arrangement were added to the majority group within each category.

For the comorbidity scale, we included the following chronic conditions: diabetes mellitus, heart disease, stroke, lung disease, and osteoporosis, all of which have been previously associated with increased frailty (Bandeen-Roche et al. 2015). Arthritis and non-skin cancer were also included in the scale. The reported value for the scale was a sum of all of the diagnoses that each subject self-reported, which were categorized as 0, 1, 2, 3, or 4 or more.

### Statistical analysis

Descriptive statistics for baseline driving status and all of the categorical baseline covariates by frailty status were examined using Chi-squared tests. To analyze the association between baseline frailty and time-varying driving status we used a generalized estimating equation analysis (GEE) specifying the log link function with a Poisson distribution (Chen et al. 2014; Hanley et al. 2003; Zou 2004). Frailty status, time point (as an ordinal variable), and their interaction were included as covariates in the model. In the unadjusted and adjusted analyses we were interested in the prevalence ratios at T1 and incidence rate ratios at each additional time point (T2-T4) for being a current non-driver among older adults who were frail at baseline.

The GEE model accounts for our longitudinally measured endpoint of driving status and our correlated data that was due to repeated measures (Hanley et al. 2003). GEE also accounted for subjects that were missing one or more of the follow-up time points at which we assessed driving status (T2, T3, and T4) (Hanley et al. 2003). Finally, GEE allowed us to account for a small proportion of individuals who were resumed drivers. These resumed drivers had been current non-drivers at one time and then became current drivers again in a follow-up round (Choi & DiNitto 2016). The above models were then adjusted for gender, age, race/ethnicity, living arrangement, dementia, and a comorbidity scale. Clustering was accounted for during the computation of standard errors. Both age and the comorbidity scale were analyzed for linearity. Age and the comorbidity scale were included in the models as ordinal variables. Time-variant covariates included living arrangement, age, and the comorbidity scale, and the remaining covariates were time-invariant. Interaction between gender and physical frailty was assessed using a cross-product term in the adjusted GEE model.

All statistical analyses were conducted with SAS Version 9.3 (SAS Institute, Cary, NC) and the final models were completed in STATA Version 14.0 (STATA Corp., College Station, TX). NHATS provides survey weights that account for selection probabilities and clustering, and are adjusted for nonresponse (Montaquila et al. 2012). These weights were developed for the purpose of producing nationally representative estimates of the older Medicare population. NHATS weighted data was used for reporting means, proportions, and measures of association.

## Results

Our analyses consisted of a study cohort of 6,288 NHATS participants with a complete baseline frailty assessment excluding never drivers at baseline. When adjusting the study sample to reflect the population using the NHATS-provided study weights, the percentage of our cohort that was frail at baseline was 15%. All of our covariates were significantly associated with frailty when they were independently tested. Almost 17% of the participants in the cohort were non-drivers at baseline, but had previously driven in their lifetime (Table 1).

**Table 1:** Characteristics of Medicare beneficiaries by frailty status at baseline, NHATS, 2011^a^

Variables	Total	Frail	Not Frail	*P-*value
Total % (N)	(6288)	15.3 (1193)	84.7 (5095)	
Driving status				<0.0001
Current driver	83.4	54.0	88.7	
Current non-driver	16.6	46.0	11.3	
Age group				<0.0001
65–69	29.4	18.2	31.5	
70–74	25.8	19.4	27.0	
75–79	19.2	17.6	19.5	
80–84	14.3	19.4	13.3	
85–89	7.9	17.2	6.2	
≥ 90	3.4	8.2	2.5	
Gender				0.0002
Female	54.0	60.0	52.9	
Male	46.0	40.0	47.1	
Race/Ethnicity				<0.0001
White non-hispanic	84.3	78.1	85.4	
Black non-hispanic	7.3	11.0	6.7	
Hispanic	5.7	8.4	5.2	
Other	2.7	2.4	2.7	
Living arrangement				0.0078
Alone	28.7	34.2	27.7	
Not Alone	71.3	65.8	72.3	
Dementia				<0.0001
Probable	6.5	19.2	4.2	
Possible or No	93.5	80.8	95.8	
Comorbidity scale				<0.0001
0	19.2	6.5	21.6	
1	30.8	17.6	33.1	
2	27.0	29.0	26.6	
3	14.3	24.1	12.6	
4 or more	8.7	22.9	6.1	

^a^Analyses of weighted data from the National Health and Aging Trends Study. Sample totals provided along with weighted percentages

Frail participants had an unadjusted incidence rate for becoming a current non-driver 3.30 (unadjusted 95% confidence interval (CI) 2.90-3.77) times that of non-frail participants at T4. There was a decrease in the incidence rate ratio for becoming a current non-driver over the T2 to T4 follow-up points, with the frailty and time interaction term significant (*p <* 0.001). When the model was adjusted for gender, race/ethnicity, living arrangement, dementia, comorbidity count and clustering, frail participants had an incidence rate of being a current non-driver 1.80 (CI 1.56–2.07) times that of non-frail participants at T4 (Table 2). The interaction between baseline frailty status and gender was tested and was not significant (*p =* 0.072).

**Table 2:** Incidence rate ratios of being a current non-driver, NHATS, 2011–2014^a^

	Unadjusted	Adjusted^b^
Time point	Incidence rate ratio^c^ (95% CI)	Incidence rate ratio^c^ (95% CI)
2011	4.01 (3.44–4.67)	2.19 (1.87–2.56)
2012	3.76 (3.27–4.31)	2.05 (1.78–2.36)
2013	3.52 (3.09–4.01)	1.92 (1.67–2.20)
2014	3.30 (2.90–3.77)	1.80 (1.56–2.07)
Frailty x Time	*p =* 0.001	*p =* 0.001

^a^Analyses of weighted data from the National Health and Aging Trends Study. Each column represents one model

^b^Multivariable Poisson Regression GEE model adjusted for gender, age, race, living arrangement, dementia, and comorbidity accounting for clustering

^c^The first row (2011) cross-sectional values are prevalence ratio estimates

## Discussion

Our analyses found that at baseline participants categorized as frail according to the frailty phenotype proposed by Fried and colleagues in 2001 had a lower driving rate relative to participants categorized as not frail. Over time, frail participants at baseline still had a higher incidence rate for becoming current non-drivers than non-frail participants at baseline, both before and after adjustment for covariates. Upon further investigation, there was not a significant interaction observed between gender and frailty status.

As a limitation of our analyses, only frailty status at baseline was utilized. Future analyses should investigate whether attenuation occurred in the current driving status and frailty relationship as time progressed. As an assessment itself, the physical frailty phenotype relies on some self-reported measures, which may not be as accurate as more objective physical measures. The comorbidity count was also dependent on self-report. Several other limitations include that we did not included employment status, socioeconomic or marital status in our analyses. This study was further limited by the scope of the NHATS driving status questions, which included a date of last time driving only in the first round. In subsequent rounds, whether drivers ever planned on resuming driving if they had not driven in the previous year was not ascertained. We note, however, that whether or not someone plans to drive again may not be a strong indicator of whether the individual will be able to or will choose to do so. We were unable to directly assess the etiology of becoming a non-driver in this study but look to future prospective older adult driving studies to move beyond associations and on to causation.

However, strengths of the study include using NHATS, a nationally representative longitudinal cohort, which provides us with a large sample size and comprehensive covariate-related questions. The frailty assessment itself includes performance-based measures. Future research on NHATS should assess frailty at multiple time points. We, chose not to impute missing frailty, as other studies (Bandeen-Roche et al. 2015) but this will probably being a necessity due to attrition in the multiple time points (DeMatteis et al. 2016). Future research designs on this topic shouldfocus on interventions and/or screening for frailty in a non-clinical setting.

## Conclusions

Frailty is still a relatively new concept in both clinical and research settings, and researchers have yet to fully reach an agreement on a frailty definition and appropriate measurements (Bergman et al. 2007). Our study posits that frailty is a concept worth exploring in a non-clinical setting and that its association with driving should be considered. As our population ages, with a projected doubling from 2010 to 2050 in older adults aged 65 and older, there will be more older drivers (Dall et al. 2013). Keeping these older adults driving as appropriate will likely be important for maintaining their health and quality of life, as being a non-driver has been demonstrated to have negative health effects for older adults (Edwards et al. 2009a; Edwards et al. 2009b; Fonda et al. 2001). As people live longer, it will be imperative to see whether these added years are enjoyable and healthy or whether they are simply an extension of lifetime. Identifying a potential association between driving status and frailty could help to identify drivers who are at risk for becoming non-drivers, as well as those who may benefit from an intervention to improve their frailty status. Keeping older adults on the road longer has the potential to help curb some of the previously mentioned adverse outcomes that are associated with driving cessation.

## Abbreviations

BMI: Body mass index
CI: Confidence interval
GEE: Generalized estimating equations
NHATS: National Health and Aging Trends Study
